# Rapid, midline retroperitoneal exposure for four-level anterior lumbar interbody fusion—technical case atlas

**DOI:** 10.1093/jscr/rjab351

**Published:** 2021-08-16

**Authors:** Rema J Malik, Mark H Falahee, Elyne N Kahn, Michael J Heidenreich, David Springstead, Abdulhameed Aziz

**Affiliations:** Department of Vascular Surgery, St. Joseph Mercy Hospital, Ypsilanti, MI, USA; Department of Vascular Surgery, St. Joseph Mercy Hospital, Ypsilanti, MI, USA; Department of Vascular Surgery, St. Joseph Mercy Hospital, Ypsilanti, MI, USA; Department of Vascular Surgery, St. Joseph Mercy Hospital, Ypsilanti, MI, USA; Department of Vascular Surgery, St. Joseph Mercy Hospital, Ypsilanti, MI, USA; Department of Vascular Surgery, St. Joseph Mercy Hospital, Ypsilanti, MI, USA

## Abstract

We describe a novel, rapid midline retroperitoneal operative technique in a patient, with multi-level degenerative scoliosis, who underwent an extensive L2-S1 anterior lumbar interbody fusion in addition to posterior instrumentation. Uniquely, our approach enables an essentially midline approach to the rectus muscle and uses the diminution of the transversalis fascia-to-peritoneum transition in the pelvis to provide expedited exposure—making it particularly helpful for ALIF exposure, retraction and intraoperative radiography. We minimize morbidity around the rectus sheath by dissecting only the medial rectus muscle and then gently, bluntly mobilizing the retroperitoneum from the deep pelvis cranially.

## INTRODUCTION

Anterior spine exposure through a retroperitoneal approach has been increasingly utilized to facilitate lumbar access for spinal fusion procedures [1, 2]. Commonly, this is done through a paramedian exposure or midline approach with full mobilization of the rectus muscle to enter the retroperitoneal plane [3].Previously described methods can take time to carefully dissect and mobilize anterior structures to delineate the retroperitoneal plane and adequately expose the spinal segment(s) of interest [6–8]. Despite its relatively wide use, a detailed, published technical description of this specific exposure, including transitions in the anterior abdominal wall with an accompanying technical atlas, does not exist. We specifically describe this rapid, midline retroperitoneal operative technique in detail as part of a case atlas in a patient, with multi-level degenerative scoliosis, who underwent an extensive L2-S1 anterior lumbar interbody fusion in addition to posterior instrumentation.

The patient has agreed to allow the authors to publish their case details and images.

## CASE REPORT

A 77-year-old female presented with decades of worsening, now intractable back pain. She had kyphosis with significant compression of her cartilaginous disk spaces throughout her lumbar spine. A longitudinal incision was fashioned between the L3-S1 segments initially using fluoroscopy to minimize incision length, with skin and subcutaneous tissues dissected down to the linea alba. The subcutaneous tissues were then cleared about 1–1.5 cm left of the midline, and we incised the anterior rectus sheath. We exposed only the medial rectus abdominis muscle, dissecting this portion off the posterior rectus sheath.

**Figure 1:**
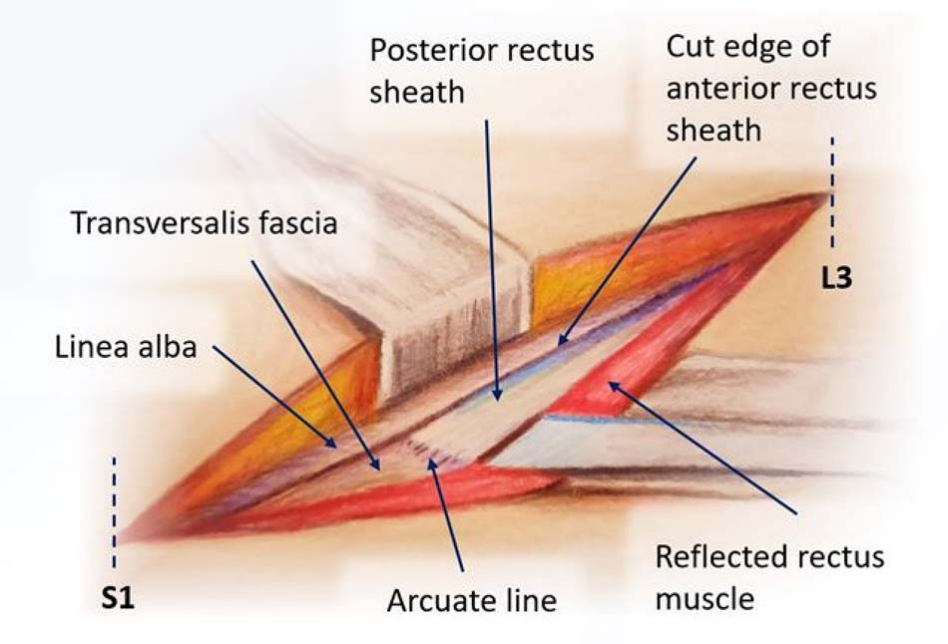
Posterior rectus sheath exposure through a midline approach.

**Figure 2:**
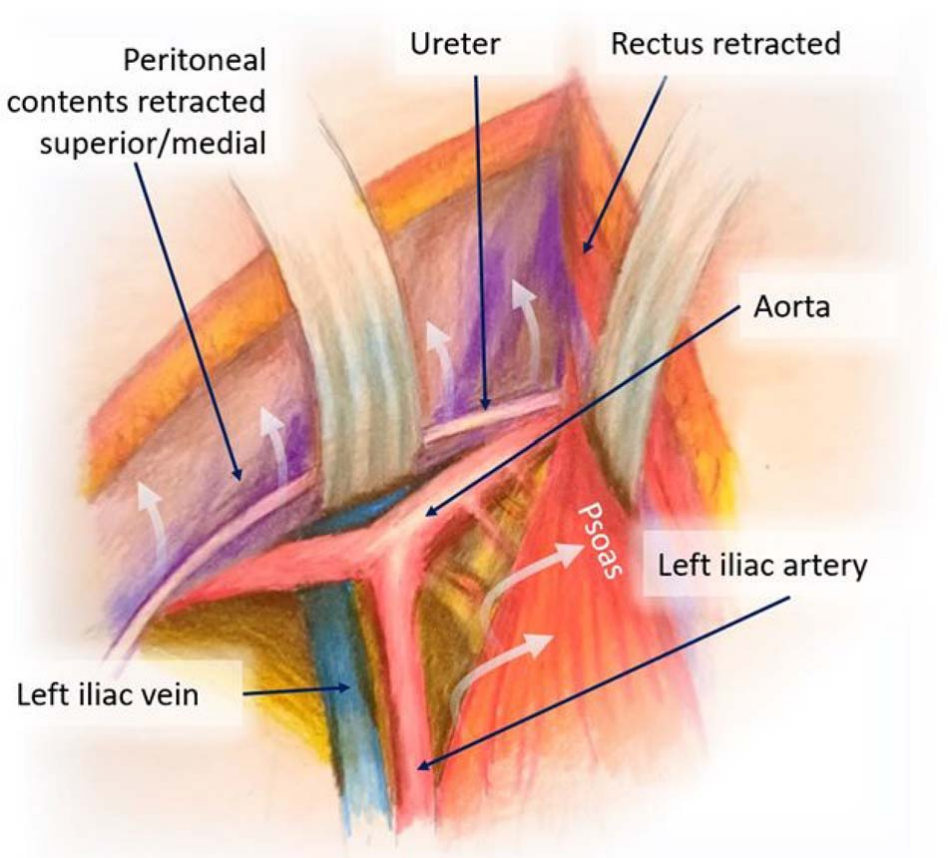
Mobilization of the peritoneal cavity and accessing the retroperitoneum.

**Figure 3:**
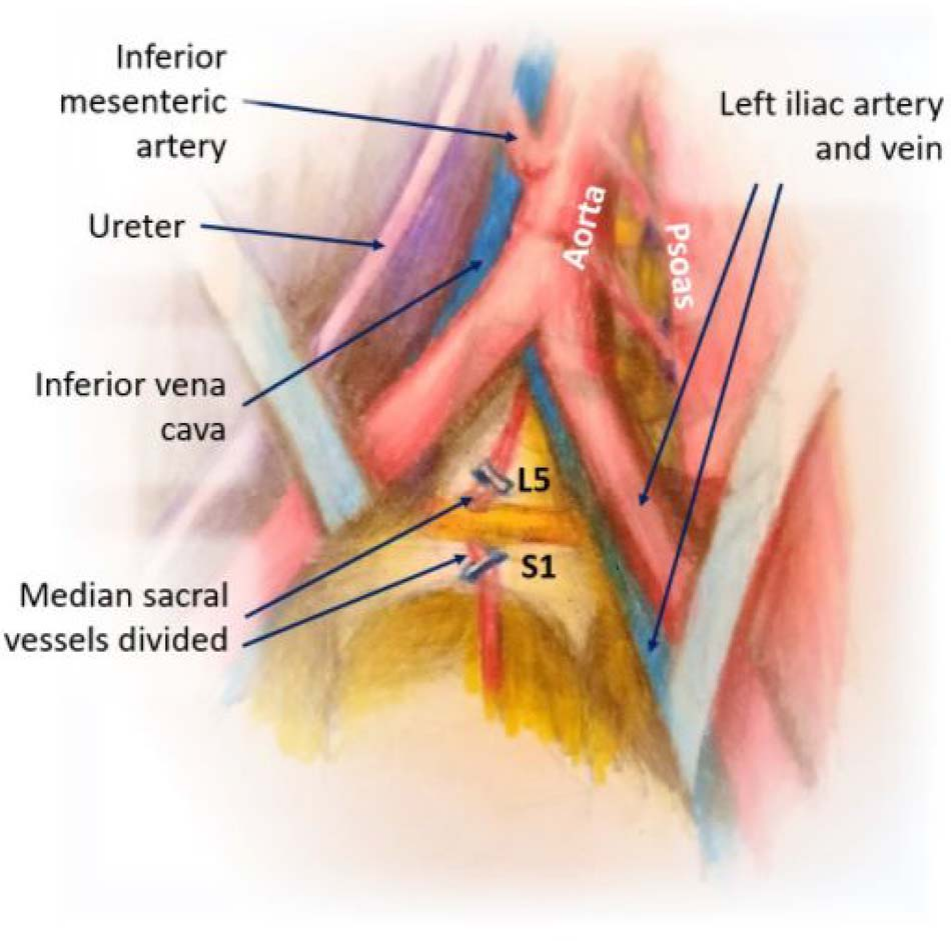
Division of iliolumbar, lumbar and median sacral vessels; exposure of L5-S1.

**Figure 4:**
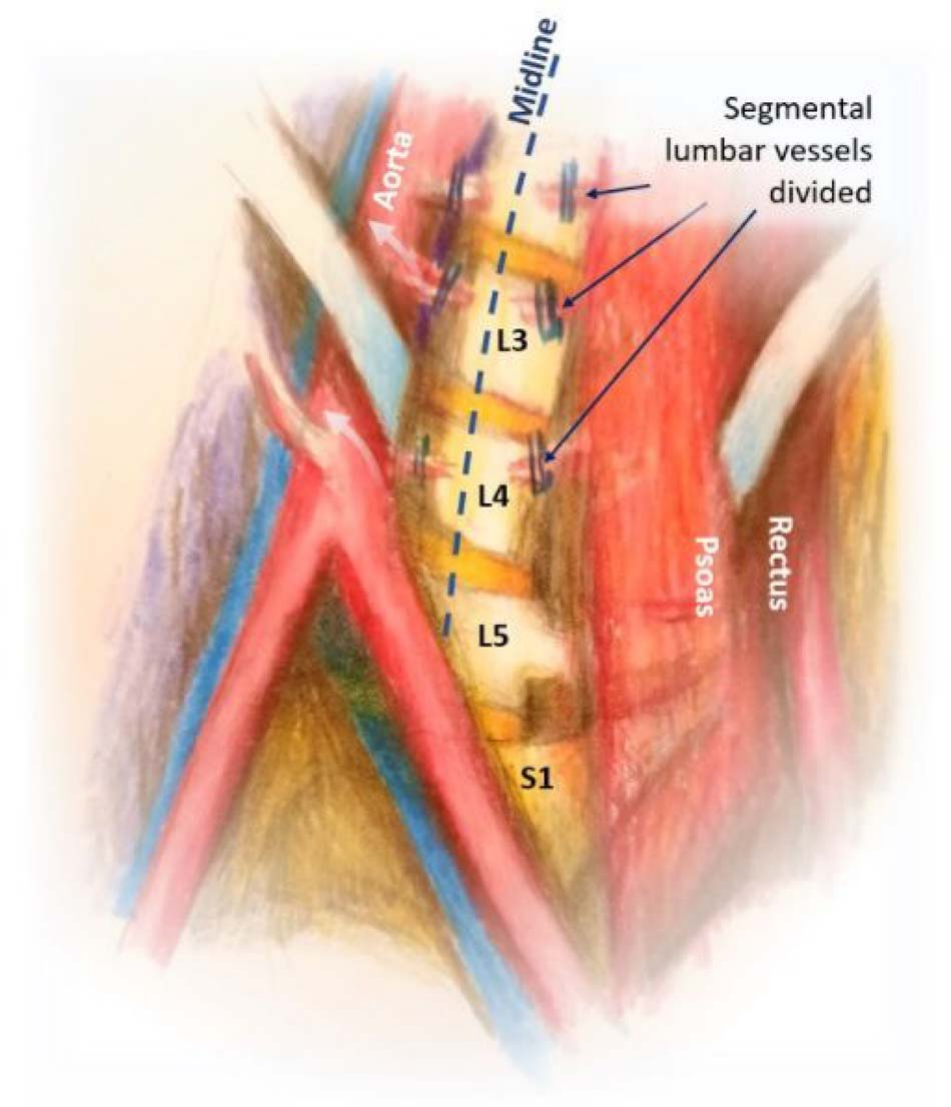
Mobilization of the aorta and IVC with exposure of the spine.

**Figure 5:**
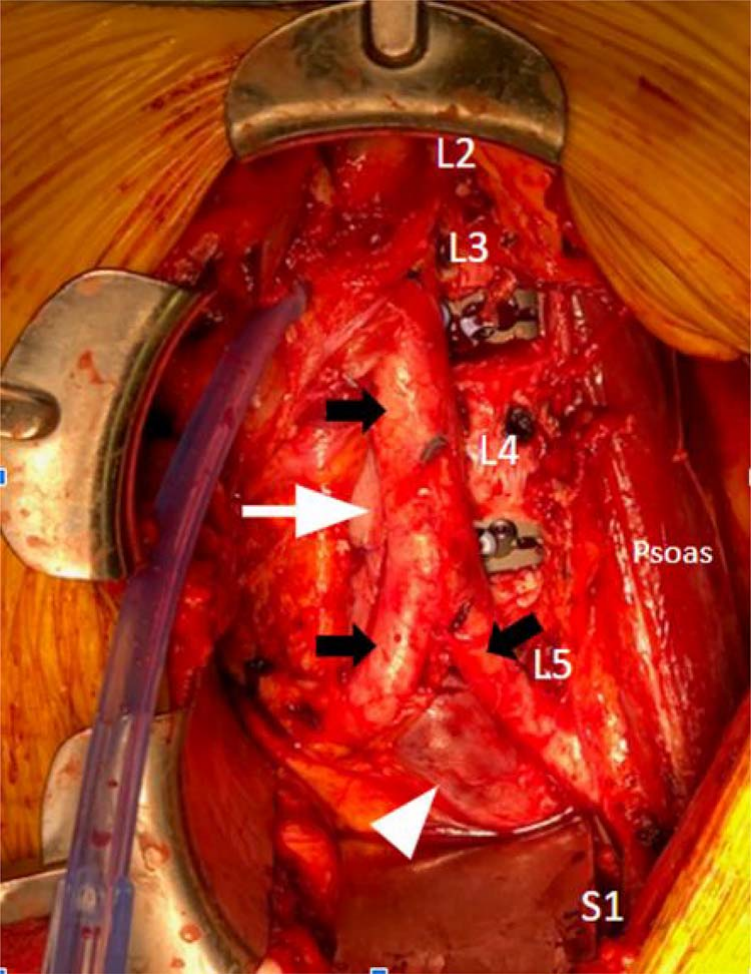
Intraoperative anterior lumbar interbody fusion L2-S1; solid black arrow, top: aorta; solid black arrows, bottom: right and left common iliac arteries; solid white arrow, top: Inferior vena cava; solid white arrow, bottom: left common iliac vein

We bluntly exposed the posterior rectus sheath with gentle upward exposure of the posterior rectus sheath (being mindful of the epigastric vessels) and could clearly visualize the arcuate line, with transversalis fascia immediately seen inferiorly (Fig. 1). We did not further mobilize the rectus muscle and were functionally midline in the abdomen. The peritoneum was carefully mobilized from the end of the incision deep in the pelvis, retracting the retroperitoneal contents superiorly and medially until the psoas muscle was easily visualized. The left ureter was easily visualized along the peritoneum and pulled toward the right side (Fig. 2). A bookwalter retractor was placed with the post on the patient’s right side, with only three retractors used: medially, laterally and superiorly. Due to her high riding pelvic brim, the IVC confluence and aortic bifurcation overlaid the L5-S1 segments. The iliolumbar vein and surrounding lumbar vessels from the aorta and inferior vena cava were carefully dissected and ligated in order to mobilize the patient’s left iliac vein toward the left side during exposure of the L5-S1 segment. Small lumbar vessels and the middle sacral vessels were clipped (Fig. 3). The remaining dissection involved pulling the abdominal aorta toward the patient’s right side. The iliac veins were all pulled toward the right side, and as the dissection continued cephalad, the aorta and IVC were gently retracted toward the right side with handheld Wiley retractors (Fig. 4). In order to facilitate exposure to the L2 level for our spine surgeons, we continued mobilization of the retroperitoneum, adjusted our retractors and clipped remaining lumbar vessels to allow handheld retraction of the aorta and IVC toward the right side. Once the anterior lumbar interbody fusion portion was completed, we irrigated the field with warm irrigation, had excellent hemostasis and the retroperitoneal contents fell back into place. The anterior rectus sheath, subdermal and skin layers were then closed. Further posterior instrumentation by the spine surgery team was done in the prone position (Fig. 5). She developed chyloperitoneum due the high dissection requiring a washout postoperative Day 9 with drain placement, which were pulled postoperative Day 8, and she was discharged to subacute rehabilitation facility. She was doing well and ambulating with significantly improved back discomfort 3 months status postsurgery.

## DISCUSSION

The use of anterior exposure of the thoracolumbar and lumbar spine has increased [9] and is most technically challenging at the L4-L5 levels, given the close proximity of iliac vessels. Zahradnik *et al*. demonstrated lower estimated blood loss, length of stay and decreased operative times in their study in which vascular surgeons provided anterior spine exposure for 159 patients [2–4]. They utilized a left paramedian fascial incision with a skin incision determined by surgeon’s preference, most often in the vertical pararectal fashion [4, 5]. Brau has described the traditional mini-open approach to the anterior spine through a 5–6 cm left lower abdominal transverse incision which begins at the midline and extends laterally [10]. This technique promotes full mobilization of the rectus muscle. We have found this has fallen out of favor due to significant retraction of the left rectus muscle toward the right side in addition to suboptimal retraction regarding spine centering during intraoperative radiography [11, 12]. In a retrospective, single center analysis of their outcomes, Manunga *et al.* report their technique for anterior lumbar interbody fusion through a midline skin incision and paramedian fascial incision [15]. Though others may be utilizing an ALIF technique staying midline entirely, to our knowledge, there are no reported data describing this technique [13, 14].

Our approach enables an essentially midline approach to the rectus muscle without full mobilization and uses diminution of the transversalis fascia-to-peritoneum transition in the pelvis to provide expedited exposure. Tactily, the retroperitoneum is easily dissected in this manner with superb midline exposure making it particularly helpful for ALIF exposure, retraction and intraoperative radiography. Though commonly used in practice, technical detail with accompanying anatomy is bereft in published literature. It is our hope this manuscript will be particularly helpful to surgeons who aim to meticulously define the anatomic planes and execute the procedure with all steps consecutively explained.

We believe that this simple yet rapid retroperitoneal technique is optimal for L5-S1 and L4-L5 exposures but can even be achieved for the rarer, multilevel exposures that we were able to achieve in the case that has been described with an excellent radiographic and technical result.
