# Correction to: Rapid, midline retroperitoneal exposure for four-level anterior lumbar interbody fusion—technical case atlas

**DOI:** 10.1093/jscr/rjac450

**Published:** 2022-09-16

This is a correction to: Rema J. Malik, Mark H. Falahee, Elyne N. Kahn, Michael J. Heidenreich, David Springstead, and Abdulhameed Aziz, Rapid, midline retroperitoneal exposure for four-level anterior lumbar interbody fusion–technical case atlas, *Journal of Surgical Case Reports*, Volume 2021, Issue 8, August 2021, rjab351, https://doi.org/10.1093/jscr/rjab351

In the originally published version of this manuscript, the order of authors was incorrect: Mark H. Falahee, Elyne N. Kahn, Michael J. Heidenreich, Abdulhameed Aziz, David Springstead, Rema J Malik

The correct order of authors is Rema J. Malik, Mark H. Falahee, Elyne N. Kahn, Michael J. Heidenreich, David Springstead, Abdulhameed Aziz

This error has been corrected online.

